# Comparative cumulative index for assessment of regression of oral homogeneous leukoplakia

**DOI:** 10.1038/s41598-026-37304-5

**Published:** 2026-01-25

**Authors:** Rakhi Chandak, Vidya Lohe, Manoj Chandak, Palak Hirani, Aditya Patel, Satyawan Singh Patel, Deepak Khobragade, Bharat Rathi

**Affiliations:** 1 SAHS, Wana, Nagpur, India; 2 Faculty of Dental Sciences, DMIHER (DU), Nagpur, India; 3 Department of Conservative Dentistry and Endodontics, SPDC, DMIHER, Nagpur, India; 4 DMIHER, Nagpur, India; 5 Datta Meghe College of Pharmacy, DMIHER (DU), Nagpur, India; 6 Dept of Rasashastra & Bhaishajya Kalpana, Mahatma Gandhi Ayurveda College Hospital and Research Centre, SalodWardha, Maharashtra India

**Keywords:** Oral conditions, Oral medicine

## Abstract

Effective treatment of oral homogeneous leukoplakia requires the use of an antioxidant with optimal properties to ensure a regression of the lesion with respect to size and colour, which is evaluated clinically and also with the help of salivary MDA levels. Despite the availability of various antioxidants, an improved standard evaluation system is needed for adjunct therapy of herbal antioxidants to widely systemically administered lycopene; a standardised evaluation system still needs to be improved. To develop a Comparative Cumulative Index (CCI) for assessment of regression of oral homogeneous leukoplakia that systematically evaluates and ranks adjunct herbal antioxidants to systemically administered lycopene based on their regression in size and colour of lesion and salivary MDA levels. CCI was developed through a structured process involving expert identification, panel discussions, and the establishment of scoring criteria. Two herbal antioxidants as an adjunct to systemically administered lycopene and lycopene alone (Groups A, B, C) were evaluated. Mean values for the core properties were calculated, and antioxidants were ranked accordingly. The total CCI for each antioxidant was computed based on the assigned scores for each property. Lesion size, lesion colour, and salivary MDA parameters all showed differences in CCI between the assessed treatment groups. Group C, with a 50% CCI. Group B’s performance, with a CCI of 41%, is highlighted by deficiencies in several properties, whereas Group A came in third with a CCI of 34%. CCI provides a comprehensive antioxidant evaluation approach to assist practitioners in making informed decisions. The significance of balancing regression in lesion size and colour as well as salivary MDA levels is highlighted by Group C’s high CCI. The results are in line with earlier studies that emphasized the significance of these traits in the treatment of oral homogeneous leukoplakia. The CCI makes it easier to compare adjunct antioxidant therapy based on empirical data and offers a reliable and equitable method of evaluating antioxidants. Future research should enhance the index and validate its use in a range of clinical contexts in order to improve treatment outcomes in oral homogeneous leukoplakia.

**Supplementary Information:**

The online version contains supplementary material available at 10.1038/s41598-026-37304-5.

## Introduction

"A predominantly white lesion or patch of the oral mucosa that may not be characterised as any other definable lesion" is how the WHO defined terminology in 1997.

Lycopene causes histological alterations in oral leukoplakia (OL), shields cells from harm, and stops dysplasia from progressing. It alters intercellular exchange junctions and prevents cell division. Lycopene may prevent cell division at G0/G1 phases by inhibiting carcinogen-induced phosphorylation of regulatory proteins such as P53 and R band ^1,2^.

The anti-inflammatory and antioxidant qualities of curcumin, a polyphenolic compound obtained from the rhizome of Curcuma longa, have been extensively studied. Because of its ability to regulate oxidative stress and inflammatory responses, curcumin has been investigated as an adjunctive agent in periodontal and endodontic applications in dentistry. However, rather than using integrated assessment frameworks, its role in oral homogeneous leukoplakia has mostly been assessed using isolated clinical outcomes ^3,4^.

Ginger has demonstrated antioxidant and anti-inflammatory activity, largely attributed to its ability to reduce oxidative stress and modulate inflammatory pathways. Preliminary clinical and experimental studies suggest potential benefits of ginger as an adjunct antioxidant in oral potentially malignant disorders. Nevertheless, comparative evaluation of ginger-containing therapies using combined clinical and biochemical outcome measures remains limited ^5,6^. Despite increasing interest in adjunct herbal antioxidants, there is currently no standardised framework that integrates clinical and biochemical parameters for comparative evaluation of therapeutic response in oral homogeneous leukoplakia.

Saliva serves as a reliable diagnostic fluid for long-term monitoring of oral diseases and oxidative stress, as reactive oxygen/nitrogen species (ROS/RNS) play a vital role in redox-dependent signalling (Valko et al., 2007). Excessive free radical production leads to oxidative stress, conducive to conditions like periodontitis and dental caries (Sies, 1997; Iannitti et al., 2012). Saliva comprises antioxidant enzymes such as superoxide dismutase, catalase, and glutathione peroxidase, contributing protection against oxidative damage (Battino et al., 2002; Amerongen & Veerman, 2002) ^7^.

Oxidative stress is caused by an imbalance that favours oxidants and can be measured using DNA oxidation indicators, oxidised proteins, and lipid peroxidation products (Maiese et al., 2010). Free radicals are reactive molecules with unpaired electrons that damage cells by changing the integrity of membranes and starting lipid peroxidation ^8^. One popular screening test for oxidative stress is the measurement of free radical activity, which is typically done by measuring the quantity of malondialdehyde (MDA) (Battino et al., 2002). One important biomarker for oxidative stress-related disorders is MDA, a hazardous aldehyde and important lipid peroxidation marker, which rises in serum during early cancer progression ^9^. The need for the additive effect of herbal antioxidants to those traditionally supplied is highlighted by the fact that oral homogeneous leukoplakia treatment can still be delayed despite the availability of many antioxidants. Presently, there is no standardised index to rank the clinical utility of the numerous available antioxidants ^10^. Practitioners have to rely on individual property assessments rather than an integrated strategy, which obscures the selection process due to the lack of a comprehensive evaluation methodology. Essential properties such as the size and colour of the lesion and salivary MDA levels are critical for the success of an antioxidant. For the therapy of oral homogeneous leukoplakia to be successful and long-lasting, these three fundamental characteristics must be harmonised ^11^. Our research intends to address this gap by creating a CCI that assesses these fundamental characteristics, helping practitioners choose the antioxidant combination therapy for clinical use.

## Material and method

All procedures were performed in compliance with applicable guidelines and regulations.

The study protocol received approval from the Institutional Ethics Committee of DMIMS (Reference No. DMIMS(DU)IEC/Dec-2019/8548; dated 16 December 2019). **Clinical trial number**—The study design and execution protocol were approved by the Institutional Ethics Committee of DMIMS (Ref No. DMIMS(DU)IEC/Dec-2019/8548; dated 16 December 2019) and were registered with the Clinical Trials Registry of India (CTRI/REF/2020/02/031,684). Written informed consent was obtained from all participants and/or their legal guardians.

### Sample size calculation for estimating a proportion

Standard formula for estimating a population proportion (Prevalence of oral homogeneous leukoplakia) as per the reference article, with absolute precision, was used for the calculation of sample size:

$${\text{n }} \ge \, \left( {{\mathrm{Z}}^{{2}} \, \times {\text{ p}}\left( {{1 } - {\text{ p}}} \right)} \right) \, /{\text{ d}}^{{2}}$$

$${\text{n }} = \, \left( {{2}.{576}^{{2}} \, \times \, 0.0{139 } \times \, \left( {{1 } - \, 0.0{139}} \right)} \right) \, / \, \left( {0.0{5}^{{2}} } \right) \, = {\text{ 37 participants}}.$$

### Development of CCI

CCI was created to evaluate and choose an antioxidant combination based on the three fundamental characteristics. The main goal of this index is to give the three recognised fundamental characteristics of an antioxidant the suitable weight. Clinicians can make well-informed decisions about the therapeutic usefulness of various antioxidants based on their unique characteristics according to this index’s systematic and quantitative evaluation.

The index development process unfolded through several meticulous steps-

*Step 1* Identification of experts.

The expert panel consisted of eight specialists in the domains of biostatistics, conservative dentistry and endodontics, Ayurvedic pharmacy, and oral medicine and radiography. A minimum of ten years of clinical or academic experience, prior publications or acknowledged expertise in oral possibly malignant illnesses or antioxidant therapy, and documented participation in institutional or national guideline formulation were the predetermined criteria used to choose experts. Expert eligibility criteria, panel size, and consensus steps were predetermined. A structured modified consensus process, consisting of two independent weighting rounds followed by a facilitated discussion, was used to resolve discrepancies and achieve unanimous agreement, guaranteeing transparency and reproducibility. A multidisciplinary expert panel, blind to study outcomes, assigned parameter weights a priori based solely on clinical relevance and biological rationale. A group of competent people was carefully selected based on their subject-matter competence. The following renowned experts were appointed as a result of this step, which sought to put together a panel of competent individuals who might offer insightful opinions to the index’s latter stages of development. When developing the CCI for evaluating the regression of oral homogeneous leukoplakia, each expert contributed a distinct set of abilities and experiences, guaranteeing a thorough and balanced viewpoint.

*Step 2* Panel discussion.

- Following a thorough panel debate, it was decided unanimously to give each of the three fundamental characteristics of the antioxidant a different weight as shown in Table 1.
  - In accordance with this agreement, the CCI for assessing the regression of oral homogeneous leukoplakia would assess the efficacy of each antioxidant uniformly across regression of salivary MDA levels and lesion size and colour. They represent complementary domains of therapeutic response in oral homogeneous leukoplakia- macroscopic disease burden, epithelial normalisation, and underlying oxidative stress, respectively- lesion size regression, lesion colour improvement, and salivary MDA reduction were chosen as essential CCI criteria. To guarantee clinical relevance, reproducibility, and feasibility, while avoiding duplication or restricted routine applicability, these parameters were determined by multidisciplinary expert consensus. Lesion size reduction was given priority as main objective result, followed by lesion colour as an epithelial healing marker, and salivary MDA as a supporting biochemical indicator. A hierarchical weighing of 50%, 30%, and 20% weighting was assigned to reflect their respective clinical value. This strategy method aimed to encourage a comprehensive and balanced examination of systemically given antioxidants, both alone and in combination with herbal antioxidants, within the CCI in order to analyse the regression of oral homogeneous leukoplakia.

**Table 1:** Weighting of core parameters used in comparative cumulative index (CCI).

Property	Weightage
Size of lesion	50%
Colour of the lesion	30%
Salivary MDA	20%

Step 3: Scoring criteria for CCI framework.

- To compare various antioxidants, mean values for each attribute were computed and noted (Group A, Group B, Group C). Scores were allocated to each antioxidant according to its mean value for each attribute. Using a formula that took into account the scores for each of the three fundamental features, the overall CCI for evaluating the regression of the oral homogeneous leukoplakia framework (in percentage) was calculated.

**Fig. 1:**
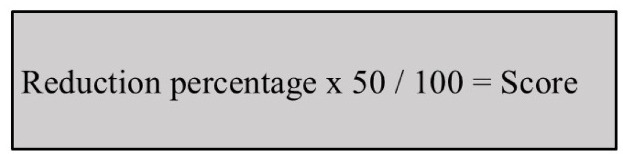
Calculation of lesion size score within the comparative cumulative index (CCI). Lesion size reduction was quantified planimetrically, expressed as a % change, and converted into a weighted score based on expert-assigned weighting criteria.

For example, when assessing the reduction percentage in lesion size, the score is determined by dividing the reduction percentage by 100 and multiplying the result by 50, as designated by the expert panel, as shown in Fig. 1.

Regarding lesion colour, a score of 0 is given for white lesions, 15 for greyish-white, and 30 when normal colour is restored, based on panel expertise as shown in Table 2.

**Table 2:** Scoring criteria for lesion colour assessment within the Comparative cumulative index (CCI).

Score	COLOUR
0	Whitish
15	Greyish white
30	Restored to normal colour

**Fig. 2:**
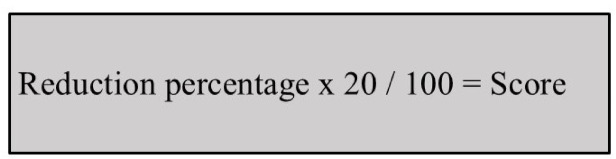
Calculation of salivary malondialdehyde (MDA) score within the comparative cumulative index (CCI). The percentage reduction in salivary MDA level was determined using the TBARS assay and converted into a weighted score according to predefined expert consensus.

Similarly, for salivary MDA level, the reduction percentage is divided by 100 and multiplied by 20, as specified by the panel, as shown in Fig. 2.

In comparing three antioxidant combinations (Group 1, Group 2, Group 3), mean values for each of the three evaluated parameters are calculated and presented in a table. Each antioxidant combination is then ranked separately for each parameter, and corresponding scores were assigned. Finally, the total CCI for evaluating the regression of oral homogeneous leukoplakia (in %) was calculated, as shown in Table 3.

**Table 3:** CCI scores across antioxidant treatment groups.

GROUPS	Anti-oxidant	INDIVIDUAL Criteria SCORE (Average)			Total Score	CCI (out of 100)
		Size of lesion	Salivary MDA	Colour of lesion		
Group I	Lycopene	Score 18	Score 2	Score 14	34	34%
Group II	Lycopene, along with curcumin gel	Score 20	Score 4	Score 17	41	41%
Group III	Lycopene, along with ginger gel	Score 25	Score 6	Score 19	50	50%

A structured modified consensus technique was adopted instead of a conventional Delphi process. In order to settle disagreements and come to a consensus decision on parameter selection, weight assignment, and scoring scales, the panel underwent two rounds of individual scoring before a facilitated group discussion. To improve transparency and reproducibility, this consensus process—including the sequential processes and technique of reaching agreement—has been thoroughly explained in the Methods section.

Prior to data collection, inter-observer reliability was established for lesion colour classification and clinical image interpretation. All clinical photos and lesion features were evaluated separately by two qualified oral medicine professionals, each with more than ten years of clinical experience. Both observers participated in a controlled calibration session using sample photos before starting the investigation. Without being aware of each other’s ratings, each observer separately rated the pictures. Cohen’s kappa statistic, which was used to gauge the degree of concordance beyond chance, was used to quantify the observers’ agreement.

A planimetric method based on graph paper that was modified from standardised wound-area evaluation techniques was used to measure the size of the lesion. A clear acetate sheet covering a calibrated 1–5 mm grid was used to sketch the lesion outline. Partial squares were only added when at least 50% of their area lay inside the boundary, and the area was calculated by counting whole squares under consistent subject positioning and lighting.

To minimise glare, controlled illumination along with cross-polarising filters, standardised intraoral photography with cheek retractors was used for evaluation of colour of lesion. A three-point index (whitish, greyish-white, and normal) was applied independently by two calibrated oral medicine specialists. Score reliability was demonstrated by Inter-observer agreement (Cohen’s kappa).

A validated TBARS (Thiobarbituric Acid Reactive Substances) test was used for Salivary MDA levels measurement. To reduce biological variability, unstimulated saliva was collected between 9:00 and 11:00 a.m. after a 90-min fast or no-intake period. To ensure analytical reliability was maintained, samples were quickly chilled, centrifuged, and examined again. Internal quality-control procedures were used to perform all experiments by a blinded biochemist.

**Fig. 3:**
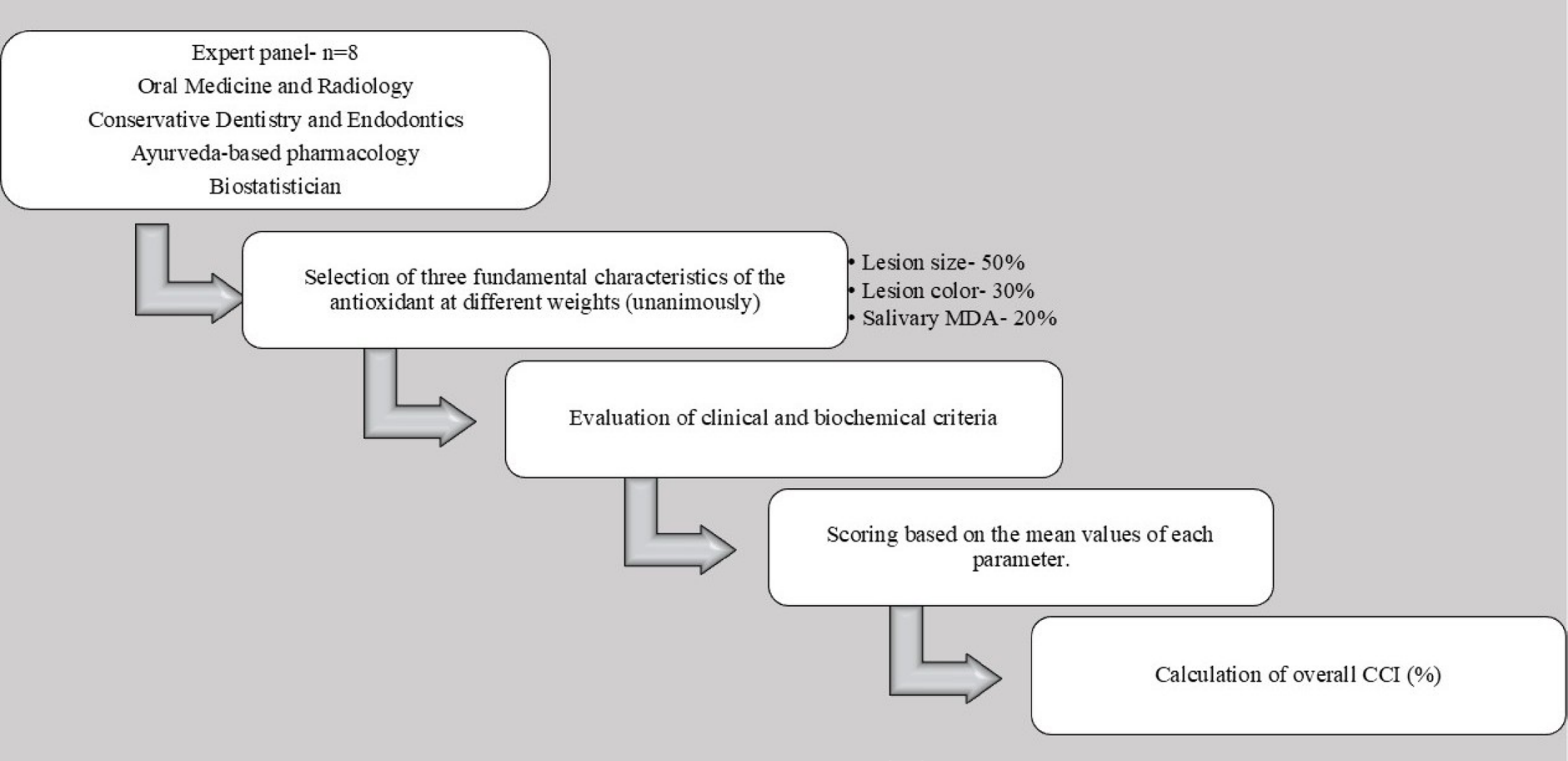
Schematic representation of development and computation of CCI. Figure outlines expert panel selection, identification and weighting of key parameters, scoring based on mean values, and calculation of overall CCI for comparative evaluation of antioxidant therapy.

The CCI is intended as a comparative evaluative framework rather than a predictive or diagnostic model; therefore, analyses requiring external gold standards (e.g., ROC/AUC) were not applicable. Lesion colour was assessed using a three-category ordinal scale (whitish, greyish-white, and normal), reflecting clinically recognised stages of epithelial change in oral homogeneous leukoplakia. Fixed scores were assigned based on expert consensus to represent the ordinal progression toward mucosal normalisation. The scale was intentionally designed to prioritise clinical relevance and reproducibility rather than fine chromatic differentiation. Summarisation of Development of CCI is depicted in Fig. 3

## Results

A thorough and quantitative assessment of the efficacy of several herbal antioxidants utilised as adjuncts to systemically administered lycopene, in comparison to lycopene alone, was offered by CCI. The highest CCI of 50% which performs better on all three core parameters, is demonstrated by Group C (Lycopene + Ginger gel). Associated with improvement of oral homogeneous leukoplakia, reflected by fewer reported side effects, early lesion size and colour regression, and a significant decrease in salivary MDA levels shown in this group.

Lesion size regression scores in Group B (Lycopene + Curcumin gel) were a bit lower than those in Group C, yet they still performed well in other parameters, having a CCI of 41%. Group A (systemically administered lycopene) came third with a CCI of 34%, indicating the need for improvements in these areas, mainly due to the notable limitations in several areas, particularly the reduction of salivary MDA levels, which are indicators of oxidative stress, and the regression of lesion size and colour.

From the consistency test among all the observers, there was agreement between both specialists. The result of the independent study of photographs of the lesion among the two specialists showed agreement, which was evident due to the measurement of Cohen’s kappa value, which was calculated to be κ = 0.82. This is, therefore, an agreement among the two specialists who demonstrated their precise and accurate categorisation of mucosal colours into the three different groups of whitish, greyish-white, and normal.

### Salivary MDA levels

#### Interpretation of pre-treatment saliva MDA levels (ηg/ml)

The MDA mean value in the Lycopene group was 4.0753 ± 0.7187, which was before the treatment and had a range of 2.85 to 5.89. The mean in the Lycopene + Curcumin group was slightly higher at 4.2673 ± 0.7015 with a range of 3.00 to 5.90, while the Lycopene + Ginger group gave a mean value of 4.2793 ± 0.7155 and a range of 2.90 to 5.95. The pre-treatment mean for all 120 patients was 4.2073 ± 0.7121. The one-way ANOVA revealed that all three groups were indistinguishable at the beginning, and there was no statistically significant difference in the levels of MDA before the treatment (F = 1.034, p = 0.358) as shown in Table 4.

**Table 4:** Baseline Saliva MDA Levels (ηg/ml) among study groups.

		N	Mean	Std. Deviation	F-value	P-value
Pre Treatment Saliva Sample (MDA levels) Î·g/ml	LYCOPENE	40	4.0753	0.71871	1.034134	0.358761
	LYCOPENE + CURCUMIN	40	4.2673	0.70152		
	LYCOPENE + GINGER	40	4.2793	0.71547		
	Total	120	4.2073	0.71214		

#### Interpretation of post-treatment saliva MDA levels (ηg/ml)

Treatment resulted in decreased MDA levels in all groups. The mean of the Lycopene group was 3.5208 ± 0.6458, ranging from 2.30 to 4.78. Lycopene + Curcumin exhibited a decline with a mean of 3.2735 ± 0.4213 (range 2.20–3.90. The reduction in this parameter was presented by the Lycopene + Ginger group with a mean value of 3.1605 ± 0.4339, followed by a range from 2.10 to 3.89. The overall mean for all groups was 3.3183 ± 0.5286. There was a statistically significant difference among 3 groups according to ANOVA after treatment (F = 5.203, p = 0.006). Therefore, the extent of reduction in MDA level was markedly different by treatment with Lycopene (p ≤ 0.05), where the maximum positive effect was noted for Lycopene + Ginger, while the smallest one was obtained after treatment with Lycopene alone, as shown in Table 5.

**Table 5:** Post-treatment Saliva MDA Levels (ηg/ml) among study groups.

		N	Mean	Std. Deviation	F-value	P-value
Post Treatment Saliva Sample (MDA levels) Î·g/ml	LYCOPENE	40	3.5208	0.64583	5.203315	0.006841
	LYCOPENE + CURCUMIN	40	3.2735	0.42133		
	LYCOPENE + GINGER	40	3.1605	0.43390		
	Total	120	3.3183	0.52858		

### Size reduction

#### Interpretation of pre-treatment size values

The Lycopene group’s mean size prior to therapy was 2.500 ± 1.127, with values ranging from 1.00 to 6.00. While the Lycopene + Ginger group had a slightly higher mean of 2.603 ± 0.829, with a minimum of 1.00 and a maximum of 4.00, the Lycopene + Curcumin group reported a similar mean of 2.513 ± 1.101, ranging between 0.50 and 6.00. The total mean of all 120 individuals was 2.538 ± 1.020.

Lesion diameters were similar prior to treatment, according to the one-way ANOVA, which showed no statistically significant difference between the three groups at baseline (F = 0.118, p = 0.888).

#### Interpretation of post-treatment size values

All groups showed a decrease in lesion size following therapy. The Lycopene group’s post-treatment size ranged from 0.620 to 3.720, with a mean of 1.550 ± 0.698. With a mean of 1.457 ± 0.638 and a range of 0.290 to 3.480, the Lycopene + Curcumin group showed a comparable decrease. The Lycopene + Ginger group showed the most decrease, with a mean post-treatment size of 1.131 ± 0.602, ranging from 0.250 to 3.000. The average size for all individuals was 1.380 ± 0.667.

The degree of size reduction varied, with the Lycopene + Ginger group showing improvement, according to the ANOVA for post-treatment values, which showed a statistically significant difference among the groups (F = 4.616, p = 0.012) as shown in Table 6.

**Table 6:** Lesion size measurements and percentage reduction following antioxidant therapy.

Size Reduction		N	Mean	Std. Deviation	F	Sig
Pre	LYCOPENE	40	2.500	1.127	0.118	0.888
	LYCOPENE + CURCUMIN	40	2.513	1.101		
	LYCOPENE + GINGER	40	2.603	0.829		
	Total	120	2.538	1.020		
Post	LYCOPENE	40	1.550	0.698	4.616	0.012
	LYCOPENE + CURCUMIN	40	1.457	0.638		
	LYCOPENE + GINGER	40	1.131	0.602		
	Total	120	1.380	0.667		
Reduction	LYCOPENE	40	37.748	0.962	11.216	< 0.01
	LYCOPENE + CURCUMIN	40	41.413	1.270		
	LYCOPENE + GINGER	40	53.525	26.961		
	Total	120	44.228	16.879		

### Colour of lesion

#### Interpretation – pre-treatment

Of the 40 individuals in each group, the Lycopene group revealed that 55.0% (22 individuals) had whitish lesions and 45.0% (18 individuals) had greyish-white lesions. 57.5% (23 individuals) and 42.5% (17 participants) of the Lycopene + Curcumin group were categorised as greyish white and whitish, respectively. 52.5% (21 individuals) and 47.5% (19 participants) of the Lycopene + Ginger group had greyish-white and whitish lesions, respectively.

Greyish-white lesions made up 37.6% of all 117 observations, while whitish lesions made up just 2.6% because three cases had missing data.

The baseline colour features were similar between the three groups, according to the chi-square test, which revealed no statistically significant variation in lesion colour distribution (χ^2^ = 1.268, p = 0.530) as shown in Table 7

**Table 7:** Baseline lesion colour distribution among study groups.

		LYCOPENE	LYCOPENE + CURCUMIN	LYCOPENE + GINGER	Chi-Square	P-value
Greyish white	Freq	18	23	21	1.268	0.530
	%	45.0%	57.5%	52.5%		
Whitish	Freq	22	17	19		
	%	55.0%	42.5%	47.5%		

#### Interpretation – post-treatment

The groups display a marked change in lesion colour after the therapy. In the Lycopene group, the percentages of patients with normal, whitish, and greyish white appearances were 32.5% (13 persons), 37.5% (15 participants), and 30.0% (12 participants), respectively. The Lycopene + Curcumin group had the following distribution of colours: greyish white, 7.5% (3 persons); whitish, 30.0% (12 persons); and a large number of people (62.5% (25 persons)) had normal mucosal colour. The results achieved by the Lycopene + Ginger group, with no one being greyish white, 5.0% being whitish, and 95.0% (38 persons) having a normal mucosal colour, which indicated almost complete colour recovery.

Among 120 cases, normal mucosa was found in 63.3% and still white was seen in 24.2%, whereas still greyish-white was observed in 12.5%.

The improvement in lesion colour also varied significantly (chi-square = 37.52, p < 0.01), such that the groups significantly differed from one another. When each treatment group was compared, the improvement in post-treatment colour normalisation was observed for Lycopene + Ginger and Lycopene + Curcumin, whereas Lycopene alone showed no improvement, as shown in Table 8

**Table 8:** Post-treatment lesion colour distribution among study groups.

		LYCOPENE	LYCOPENE + CURCUMIN	LYCOPENE + GINGER	Chi-Square	P-value
Greyish white	Freq	12	3	0	37.52	< 0.01
	%	30.0%	7.5%	0.0%		
Whitish	Freq	15	12	2		
	%	37.5%	30.0%	5.0%		
		13	25	38		
		32.5%	62.5%	95.0%		

This result supports existing research and emphasises the importance of a well-balanced effect on these key features to ensure optimal therapeutic outcomes. Group C showed better relative performance for practitioners treating patients with oral homogeneous leukoplakia based on the high antioxidant activity and the considerable decrease in the salivary MDA levels, and the noticeable regression of the lesion size and colour.

It appears that some areas may require extra optimisation, although Group B remains a viable treatment choice.

The above results underscore the never-ending need for innovative pharmacist-formulated antioxidants to improve their potential therapeutic indices beyond the current difficulties.

The results of Cronbach’s alpha test revealed an acceptable internal consistency among the variables of lesion size reduction, lesion colour improvement, and salivary MDA reduction, thus validating their composite formation. Pearson correlation test showed a low to moderate correlation between lesion size reduction, lesion colour improvement, and salivary MDA reduction, and all were below the collinearity limits (r < 0.7). Variance inflation factor analysis further confirmed independence of parameters, with all VIF values below 2. These findings indicate that each component contributes unique information to the composite index without redundancy.

Bootstrap resampling (1,000 iterations) demonstrated consistent CCI score distributions and preserved the relative ranking of antioxidant groups, confirming internal stability of the index within the study population.

“Validation of the Comparative Cumulative Index (CCI)”

Internal consistency of the Comparative Cumulative Index (CCI) was evaluated using Cronbach’s alpha to assess whether lesion size reduction, lesion colour improvement, and salivary malondialdehyde (MDA) reduction coherently represent a single composite construct reflecting therapeutic regression. The analysis demonstrated acceptable internal consistency (Cronbach’s α ≥ 0.70), indicating that the three parameters are sufficiently interrelated to justify their integration into a composite index without evidence of redundancy.

To evaluate robustness of the weighting scheme, a sensitivity analysis was performed by varying the assigned weights of lesion size, lesion colour, and salivary MDA reduction by ± 10% while maintaining the total weight at 100%. Across all tested scenarios, the relative CCI scores and ranking of treatment groups remained unchanged, demonstrating that the index is structurally robust and not disproportionately driven by a single parameter.

Internal stability of the CCI was assessed using bootstrap resampling with 1,000 iterations. In each resampled dataset, CCI scores were recalculated, and group rankings were examined. The relative ordering of antioxidant groups remained consistent across bootstrap samples, indicating that the index demonstrates stable performance and that its conclusions are not dependent on random sampling variation within the study cohort.

Potential multicollinearity among lesion size reduction, lesion colour improvement, and salivary MDA reduction was assessed using Pearson correlation coefficients and variance inflation factors (VIFs). All correlation coefficients remained below established collinearity thresholds, and VIF values were < 2, confirming that each parameter contributes independent information to the composite index.

## Discussion

In the field of oral medicine, studies evaluating CCI on the regression of oral homogeneous leukoplakia related to antioxidant therapy aim to evaluate and provide an assessment for clinical improvement of such lesions. The effectiveness of different antioxidants and their combinations in the treatment of oral homogenous leukoplakia can be estimated by using this detailed and standardised way. Basically, the CCI employed in this research work on the regression of oral homogeneous leukoplakia was developed through the intensive survey of the existing literature, as well as through the expertise of specialists working in this particular area. Though there was research carried out on identifying the most significant factors of effectiveness for antioxidants, it was the opinion of the specialist group that was considered as the final standard for determination of the index. In order for the CCI to encompass scientific facts as well as medical expertise, it was absolutely integral to include the participation of these specialists so as to help determine the most significant criteria for monitoring. There was also an enhancement of relevance and applicability through being grounded on scientific facts as well as medical expertise.

A standardised procedure was developed to calculate the mean value for each of the key features in order to facilitate an assessment of the individual effects of each antioxidant. This method ensures that all forms of antioxidant therapy are judged in an identical way, and an equal comparison between all features is feasible. The CCI is a useful means of assessing the effectiveness of treatment in oral homogeneous leukoplakia because it contains a variety of key features, like the size of the lesion, its colour, and MDA in the salivary fluids.

By neutralising reactive oxygen species (ROS), reestablishing redox equilibrium, and lowering chronic inflammation, antioxidants inhibit these effects and promote lesion reduction and epithelial repair. Strong free radical scavenging abilities seen in herbal antioxidants, including lycopene, curcumin, and ginger, improve cellular repair processes and prevent oxidative damage. Clinical evidence has demonstrated that antioxidant therapy causes a discernible reduction in lesion size over time, especially when combined with systemic medications ^12^.

Our results are consistent with earlier research that found lycopene and ginger to have higher antioxidant qualities when combined. The effectiveness of antioxidant therapy, which remains non-invasive and successful in treating Oral Homogeneous Leukoplakia, has been supported by clinical evidence suggesting that improvements in mucosa colour are often one of the early signs predictive of success ^13^.

Acting as scavengers for reactive oxygen species (ROS) and helping to stabilise cellular membranes, antioxidants can prevent this from happening and can significantly lower the production of MDA. Superior antioxidant properties, as seen in herbs such as lycopene, curcumin, and ginger, can restore the salivary level of MDA to normal, signifying a positive oxidation status. Together with the positive oxidation status, the reduction of MDA can also indicate positive clinical responses, manifested by the reduction of the size and colour of the lesion. Salivary MDA concentrations are a non-invasive and measurable marker known to provide crucial data regarding the biochemical role of antioxidant treatment, thereby proving its effectiveness for the successful treatment of oral homogeneous leukoplakia ^14^.

Consequently, we were able to focus on major attributes that may be quantified and directly influence the treatment efficacy of oral homogeneous leukoplakia, including the size and pigmentation of the lesion, as well as salivary MDA levels. To ensure a better perspective on the efficacy and long-term correlations of Herbal Antioxidants in the treatment of oral homogeneous leukoplakia, it would be desirable that these major considerations should be focused on in future studies.

The statistical validation that followed, which showed internal consistency, minimal collinearity, and stability, supports the idea that the final parameter set reflects methodological rigour rather than subjective preference, even though expert consensus was employed for parameter selection.

The CCI was developed as a comparative evaluative index intended to integrate multiple clinically and biologically relevant outcomes into a single summary measure. It was not designed as a diagnostic or predictive classifier. Consequently, analyses that require a binary or categorical reference standard—such as receiver operating characteristic (ROC) curves, area under the curve (AUC) estimation, or agreement statistics—were not applicable, as no validated gold-standard definition of “treatment success” exists for oral homogeneous leukoplakia. Instead, validation focused on internal consistency, weighting robustness, stability, and parameter independence, which are appropriate for the construction and assessment of a composite evaluative index.

Although advanced digital image analysis techniques such as CIELAB colour space evaluation offer superior objectivity, their requirement for specialised imaging infrastructure limits routine clinical applicability. The present study, therefore, employed a calibrated, clinically interpretable colour scoring system. Future studies should integrate digital colourimetric methods to further refine and externally validate the colour component of the CCI.

## Limitations

In assessing the outcome of the study, it is essential to consider the limitations of the study. The nature of lesion as well as patient factors can differ from one population of patients to another. The study is conducted in only one centre. The CCI cannot be extended to other antioxidant preparations because here only two different supplementary antioxidant herbs were tested together with the lycopene.

Despite the regular and controlled nature of expert consensus, subjective bias may still be present in the variable weighting. Furthermore, although being standardised by calibrated photography and planimetric tracing, lesion size and colour judgments are still subject to observer-dependent variation. Despite regulated collection and laboratory procedures, biological variability may still have an impact on salivary MDA results. Finally, the CCI underwent internal validation only; external validation across multi-centre cohorts and with additional antioxidant combinations is necessary to confirm its reproducibility, robustness, and wider clinical utility.

The study was not randomised or blinded, the sample size was limited, and substantial inter-individual variability was observed. Furthermore, the CCI has undergone internal validation only and lacks external validation. Consequently, the results should be viewed as hypothesis-generating rather than practice-changing, and further randomised, blinded, and multicentric studies are required before clinical recommendations can be made.

## Conclusion

Based on key therapeutic characteristics, the CCI developed in the present study provides a comprehensive and impartial model for evaluation and ranking of herbal medicinal antioxidant combinations. Through salivary MDA level reductions and lesion size and colour regressions, this index permits a comprehensive assessment of antioxidant activity. Group C’s comparatively higher performance within the study cohort underscores the importance of considering diverse perspectives in the selection of an adjuvant antioxidant treatment. Future studies should concentrate on improving this index even further, adding more assessment criteria, and confirming that it can be used in clinical settings. By continuously enhancing the criteria used to evaluate adjunct antioxidant therapies, we can improve both the quality and effectiveness of treatment strategies for oral homogeneous leukoplakia.

## Supplementary Information

Below is the link to the electronic supplementary material.

Supplementary Material 1

## Data Availability

"Data is provided within the supplementary information files.”

## Abbreviations

CCI: Clinical Cumulative Index for assessment of regression of oral homogeneous leukoplakia
MDA: Malondialdehyde
ROS: Reactive oxygen species
COX-2: Cyclooxygenase -2
OL: Oral Leukoplakia
