# Supplementary material for: Comparative cumulative index for assessment of regression of oral homogeneous leukoplakia
Source: Sci Rep. 2026 Jan 25;16:6200. doi: 10.1038/s41598-026-37304-5 (PMC12905322; doi:10.1038/s41598-026-37304-5)
Supplement: Supplementary file 1 — Supplementary Material 1 [file 41598_2026_37304_MOESM1_ESM.docx]

|  |  | N | Mean | Std. Deviation | Std. Error | 95% Confidence Interval for Mean | | | Minimum | Maximum | F-value | P-value |
| --- | --- | --- | --- | --- | --- | --- | --- | --- | --- | --- | --- | --- |
|  |  |  |  |  |  | Lower Bound | Upper Bound | |  |  |  |  |
| Pre Treatment Saliva Sample (MDA levels) Î·g/ml | LYCOPENE | 40 | 4.0753 | 0.71871 | 0.11364 | 3.8454 | | 4.3051 | 2.85 | 5.89 | 1.034134 | 0.358761 |
|  | LYCOPENE+CURCUMIN | 40 | 4.2673 | 0.70152 | 0.11092 | 4.0429 | | 4.4916 | 3.00 | 5.90 |  |  |
|  | LYCOPENE+GINGER | 40 | 4.2793 | 0.71547 | 0.11313 | 4.0504 | | 4.5081 | 2.90 | 5.95 |  |  |
|  | Total | 120 | 4.2073 | 0.71214 | 0.06501 | 4.0785 | | 4.3360 | 2.85 | 5.95 |  |  |

**Table 1: Baseline unstimulated salivary MDA levels (ηg/ml) across the three treatment groups before intervention.**

|  | | N | Mean | Std. Deviation | Std. Error | 95% Confidence Interval for Mean |  | Minimum | Maximum | F-value | P-value |
| --- | --- | --- | --- | --- | --- | --- | --- | --- | --- | --- | --- |
|  |  |  |  |  |  | Lower Bound | Upper Bound |  |  |  |  |
| Post Treatment Saliva Sample (MDA levels) Î·g/ml | LYCOPENE | 40 | 3.5208 | 0.64583 | 0.10212 | 3.3142 | 3.7273 | 2.30 | 4.78 | 5.203315 | 0.006841 |
|  | LYCOPENE+CURCUMIN | 40 | 3.2735 | 0.42133 | 0.06662 | 3.1388 | 3.4082 | 2.20 | 3.90 |  |  |
|  | LYCOPENE+GINGER | 40 | 3.1605 | 0.43390 | 0.06861 | 3.0217 | 3.2993 | 2.10 | 3.89 |  |  |
|  | Total | 120 | 3.3183 | 0.52858 | 0.04825 | 3.2227 | 3.4138 | 2.10 | 4.78 |  |  |

**Table 2: Post-treatment salivary MDA levels (ηg/ml) following antioxidant therapy.**

| Size Reduction | | N | Mean | Std. Deviation | Std. Error | 95% Confidence Interval for Mean |  | Minimum | Maximum | F-value | P-value |
| --- | --- | --- | --- | --- | --- | --- | --- | --- | --- | --- | --- |
|  |  |  |  |  |  | Lower Bound | Upper Bound |  |  | F | Sig. |
| Pre | LYCOPENE | 40 | 2.500 | 1.127 | 0.178 | 2.140 | 2.860 | 1.000 | 6.000 | 0.118 | 0.888 |
|  | LYCOPENE+ CURCUMIN | 40 | 2.513 | 1.101 | 0.174 | 2.161 | 2.864 | 0.500 | 6.000 |  |  |
|  | LYCOPENE+ GINGER | 40 | 2.603 | 0.829 | 0.131 | 2.337 | 2.868 | 1.000 | 4.000 |  |  |
|  | Total | 120 | 2.538 | 1.020 | 0.093 | 2.354 | 2.723 | 0.500 | 6.000 |  |  |
| Post | LYCOPENE | 40 | 1.550 | 0.698 | 0.110 | 1.327 | 1.773 | 0.620 | 3.720 | 4.616 | 0.012 |
|  | LYCOPENE+ CURCUMIN | 40 | 1.457 | 0.638 | 0.101 | 1.253 | 1.661 | 0.290 | 3.480 |  |  |
|  | LYCOPENE+ GINGER | 40 | 1.131 | 0.602 | 0.095 | 0.939 | 1.324 | 0.250 | 3.000 |  |  |
|  | Total | 120 | 1.380 | 0.667 | 0.061 | 1.259 | 1.500 | 0.250 | 3.720 |  |  |
| Reduction | LYCOPENE | 40 | 37.748 | 0.962 | 0.152 | 37.440 | 38.055 | 35.500 | 39.500 | 11.216 | <0.01 |
|  | LYCOPENE+ CURCUMIN | 40 | 41.413 | 1.270 | 0.201 | 41.006 | 41.819 | 39.000 | 44.000 |  |  |
|  | LYCOPENE+ GINGER | 40 | 53.525 | 26.961 | 4.263 | 44.902 | 62.148 | 50.000 | 92.000 |  |  |
|  | Total | 120 | 44.228 | 16.879 | 1.541 | 41.177 | 47.279 | 50.000 | 92.000 |  |  |

**Table 3: Pre- and post-treatment lesion size measurements and percentage reduction across treatment groups.**

|  | | LYCOPENE | LYCOPENE+ CURCUMIN | LYCOPENE+ GINGER | Total | Chi-Square | P-value |
| --- | --- | --- | --- | --- | --- | --- | --- |
|  |  |  |  |  |  |  |  |
| Greyish white | Freq | 18 | 23 | 21 | 44 | 1.268 | 0.530 |
|  | % | 45.0% | 57.5% | 52.5% | 37.6% |  |  |
| Whitish | Freq | 22 | 17 | 19 | 3 |  |  |
|  | % | 55.0% | 42.5% | 47.5% | 2.6% |  |  |
| Total | Freq | 40 | 40 | 40 | 117 |  |  |
|  | % | 100.0% | 100.0% | 100.0% | 100.0% |  |  |

**Table 4: Baseline distribution of lesion colour (whitish and greyish-white) across treatment groups**

|  | | LYCOPENE | LYCOPENE+ CURCUMIN | LYCOPENE+ GINGER | Total | Chi-Square | P-value |
| --- | --- | --- | --- | --- | --- | --- | --- |
|  |  |  |  |  |  |  |  |
| Greyish white | Freq | 12 | 3 | 0 | 15 | 37.52 | <0.01 |
|  | % | 30.0% | 7.5% | 0.0% | 12.5% |  |  |
| Whitish | Freq | 15 | 12 | 2 | 29 |  |  |
|  | % | 37.5% | 30.0% | 5.0% | 24.2% |  |  |
|  |  | 13 | 25 | 38 | 76 |  |  |
|  |  | 32.5% | 62.5% | 95.0% | 63.3% |  |  |
| Total | Freq | 40 | 40 | 40 | 120 |  |  |
|  | % | 100.0% | 100.0% | 100.0% | 100.0% |  |  |

**Table 5: Post-treatment distribution of lesion colour (whitish, greyish-white, and normal) across treatment groups**
